# Predicting the Toxicity In Silico of the Aqueous Extract of *Chiranthodendron pentadactylon* Flowers. Experimental Evaluation In Vivo

**DOI:** 10.3390/ph19091354

**Published:** 2026-08-26

**Authors:** Oscar Salvador Barrera-Vázquez, Gil Alfonso Magos-Guerrero, Juan Luis Escobar-Ramírez, Rubén San Miguel-Chávez, Maira Huerta-Reyes

**Affiliations:** 1 Department of Pharmacology, Faculty of Medicine, University National Autonomous of Mexico (UNAM), Mexico City 04510, Mexico; osbarrerav@comunidad.unam.mx (O.S.B.-V.); re-harakhty@hotmail.com (J.L.E.-R.); 2 Posgrado en Botánica, Campus Montecillo, Colegio de Posgraduados, México-Texcoco Highway km 35.6, Texcoco 56230, Mexico; sanmi@colpos.mx; 3 Unidad de Investigación Médica en Enfermedades Nefrológicas, Hospital de Especialidades “Dr. Bernardo Sepúlveda Gutiérrez”, Centro Médico Nacional Siglo XXI, Instituto Mexicano del Seguro Social, Cuauhtémoc, Ciudad de México 06720, Mexico; chilanguisima@yahoo.com

**Keywords:** computational toxicology, cheminformatics, medicinal plants, aqueous extract, *Chiranthodendron pentadactylon*, median lethal dose

## Abstract

*Chiranthodendron pentadactylon* Larreat (“flor de manita”) is traditionally used for gastrointestinal, cardiovascular, and neurological disorders. **Objective:** This study comprehensively integrated phytochemical characterization, in silico toxicological profiling, an exposure-informed Integrative Toxicity Prediction Model (ITPM), uncertainty analysis, and acute testing of a fresh flower aqueous extract (FFAE). **Methods:** Thirty-six phytochemicals were screened for hepatotoxicity, nephrotoxicity, Ames mutagenicity, carcinogenicity, hERG inhibition, reproductive-effects alerts, and acute oral toxicity. Five organ- or effect-specific endpoints were integrated into an exploratory Toxicological Alert Score (TAS), whereas reproductive alerts and predicted LD_50_ were reported separately. Seventeen compounds quantified in the FFAE were compared with a 20-compound literature-informed profile through 50,000 Monte Carlo iterations. Male CD-1 mice received a single FFAE dose of 300–5000 mg/kg and were observed for 14 days. **Results:** Positive predictions occurred for hERG inhibition in 33 compounds (91.7%), hepatotoxicity in 29 (80.6%), nephrotoxicity and carcinogenicity in 28 each (77.8%), and mutagenicity in 21 (58.3%). Six compounds had predicted LD_50_ values ≤ 1000 mg/kg. The proportional ITPM toxicity-evidence index was 6.91% for the experimental profile and 19.34% for the literature-informed profile. Monte Carlo means were 6.98% (95% uncertainty interval, 5.65–8.44%) and 19.32% (16.76–22.37%), respectively. No mortality, overt clinical signs, or treatment-related body weight differences occurred up to 5000 mg/kg. **Conclusions:** Compound-level predictions identified priorities for confirmatory testing, while the extract-specific model yielded a lower comparative toxicity-evidence proportion, and the animal study showed low overall acute toxicity under the evaluated conditions. These findings do not establish general or long-term safety, calibrated risk probabilities, or biological neutralization of toxicological liabilities.

## 1. Introduction

The use of medicinal plants remains widespread worldwide [1,2], particularly in regions with substantial biological diversity and ethnobotanical richness, such as Mexico, where traditional medicine is an important component of healthcare [3]. Mexico harbors approximately 23,424 vascular plant species, including nearly 5000 endemic species. Approximately 4500 species are medicinal, while around 3000 are registered in the herbarium of the Mexican Institute of Social Security (IMSS) [4]. Nevertheless, pharmacological information has reportedly been generated for only approximately 5% of the medicinal species documented in Mexico [1,2,3,4,5], limiting the assessment of their efficacy and safety.

The perception that “natural” is synonymous with “safe” [6,7] may encourage the use of medicinal plants containing constituents associated with acute or long-term toxicity [8]. Hepatotoxicity, nephrotoxicity, neurotoxicity, hematological alterations, and mortality have been associated with incorrectly identified, inadequately prepared, or improperly administered plant products [9,10,11,12,13]. Rigorous approaches are therefore needed to identify potential hazards and assess their safety [13]. In silico methods, including molecular modeling, ADME/Tox prediction, and ligand–target simulations, provide rapid, cost-effective tools for prioritizing compounds for experimental evaluation and may reduce animal use and experimental resources [14,15,16]. However, conventional in silico toxicology evaluates compounds as isolated entities. Extrapolating these predictions to medicinal preparations may provide an incomplete representation of the toxicological behavior of whole extracts. Aqueous extracts are complex mixtures in which each constituent’s contribution depends on its intrinsic properties, concentration, extraction yield, bioavailability, metabolism, and potential interactions [17,18,19]. This means a predicted alert for one compound does not confirm the extract’s toxicity.

This distinction is consistent with frameworks that separate hazard identification from exposure assessment [20,21]. Hazard is the intrinsic capacity to cause harm, whereas risk also depends on the magnitude, route, frequency, and duration of exposure. For plant extracts, relevant variables include constituent abundance, extraction recovery, administered dose, and bioavailability. Frameworks for combined exposure recommend problem formulation, tiered assessment, evidence weighting, and uncertainty characterization [21,22,23], while Integrated Approaches to Testing and Assessment combine computational, physicochemical, and experimental information to support hazard characterization [24]. These principles have rarely been adapted to phytochemical complexes containing both predicted toxicological and protective properties.

Antioxidant, anti-inflammatory, cytoprotective, antimutagenic, anti-genotoxic, and xenobiotic defense activities were selected as protective attributes because they may limit mechanisms involved in toxic injury [17,18,19,25,26]. Combined, they help cells stay healthy by reducing oxidative and inflammatory damage, preventing mutations, and aiding the body in expelling foreign invaders. They were incorporated as separate biological attributes, not as evidence of toxicological neutralization. A predicted protective activity cannot be interpreted as offsetting a toxicological alert unless both are mechanistically related, occur at biologically relevant concentrations, and are shown under comparable exposure conditions. The resulting scores must therefore be interpreted as comparative indices, not probabilities of toxicity, protection, or safety.

The Integrative Toxicity Prediction Model (ITPM) was developed as an exploratory, evidence-weighted, mixture-level computational framework. It was designed to compare phytochemical scenarios and identify constituents or evidence domains warranting further investigation; it was not designed to estimate individual or population risk, predict clinical adverse-event probabilities, establish a safe dose, or replace experimental toxicological assessment. Rather than interpreting each phytochemical separately, it evaluates the identified constituents collectively and weights their toxicological and protective predictions using exposure-informed variables, including predicted bioavailability and extraction yield. The resulting values estimate relative model-derived contributions to the extract profile, not measured systemic exposures or experimentally quantified effects. The model also does not demonstrate pharmacokinetic or pharmacodynamic interactions. Compared with live animal results, it gives a basic check of whether the findings make biological sense, but it is not complete proof. Full validation needs different samples, data, exposure methods, and toxicity tests.

*Chiranthodendron pentadactylon* Larreat, known in Mexico as “flor de manita” and in Nahuatl as “macpalxochitl”—from *macpalli* (hand) and *xochitl* (flower)—is endemic to Mexico [27]. Its flowers have been used since pre-Hispanic times for chronic ulcers, ocular pain, and inflammation and are currently used for heart conditions, epilepsy, diarrhea, and dysentery [28,29,30,31]. Flower extracts have exhibited anticholinergic, spasmolytic, antiprotozoal, antibacterial, vasorelaxant, antihypertensive, and antisecretory activities [27,28,29,30,31,32,33,34,35,36].

Despite these uses, the toxicological profile of *C. pentadactylon* flowers remains unexplored. The only toxicological evidence identified in the databases and sources searched was an academic thesis suggesting that an aqueous flower extract may produce toxic effects [37]. No additional toxicological studies were identified, and these findings have not been independently replicated. Current data do not confirm reproducible toxicity from the whole-flower extract or identify its toxic components, safe exposure levels, or underlying mechanisms. This study is the first to combine information on the plant’s chemical components, predictions of compound toxicity and protection, exposure-related details, and an acute oral toxicity study.

Our hypothesis is that integrating the predicted toxicological and protective properties of the identified constituents, along with bioavailability and extraction yield, will generate a model-derived, exposure-based profile that differs from an unweighted interpretation of alerts at the level of isolated compounds. Accordingly, the ITPM was applied to the phytochemical mixture, and the results were interpreted in conjunction with observations of mortality and overt toxicity in male CD-1 mice following oral administration of the extract. This model is designed to highlight and rank toxicological risks, not to confirm safety levels or to prove that alerts are resolved.

## 2. Results

### 2.1. Extraction Yield and HPLC Characterization of the FFAE

The fresh flower aqueous extract (FFAE) was obtained with a dry extraction yield of 4.45% (6.0 g from 135 g of fresh flowers). HPLC analysis detected and quantified 17 metabolites in the extract and measured their total amount at 132.73 mg/g. Rosmarinic acid was the most abundant constituent of the phytocomplex (100.91 mg/g; 76.03%), followed by catechin (15.20 mg/g; 11.45%), oleanolic acid (4.60 mg/g; 3.47%), stigmasterol (3.51 mg/g; 2.64%), cyanidin 3-O-glucoside (3.42 mg/g; 2.58%), phloretin (2.09 mg/g; 1.57%), and α-amyrin (1.18 mg/g; 0.89%). The remaining compounds individually represented less than 0.5% of the total quantified phytochemical content.

**Figure 1:**
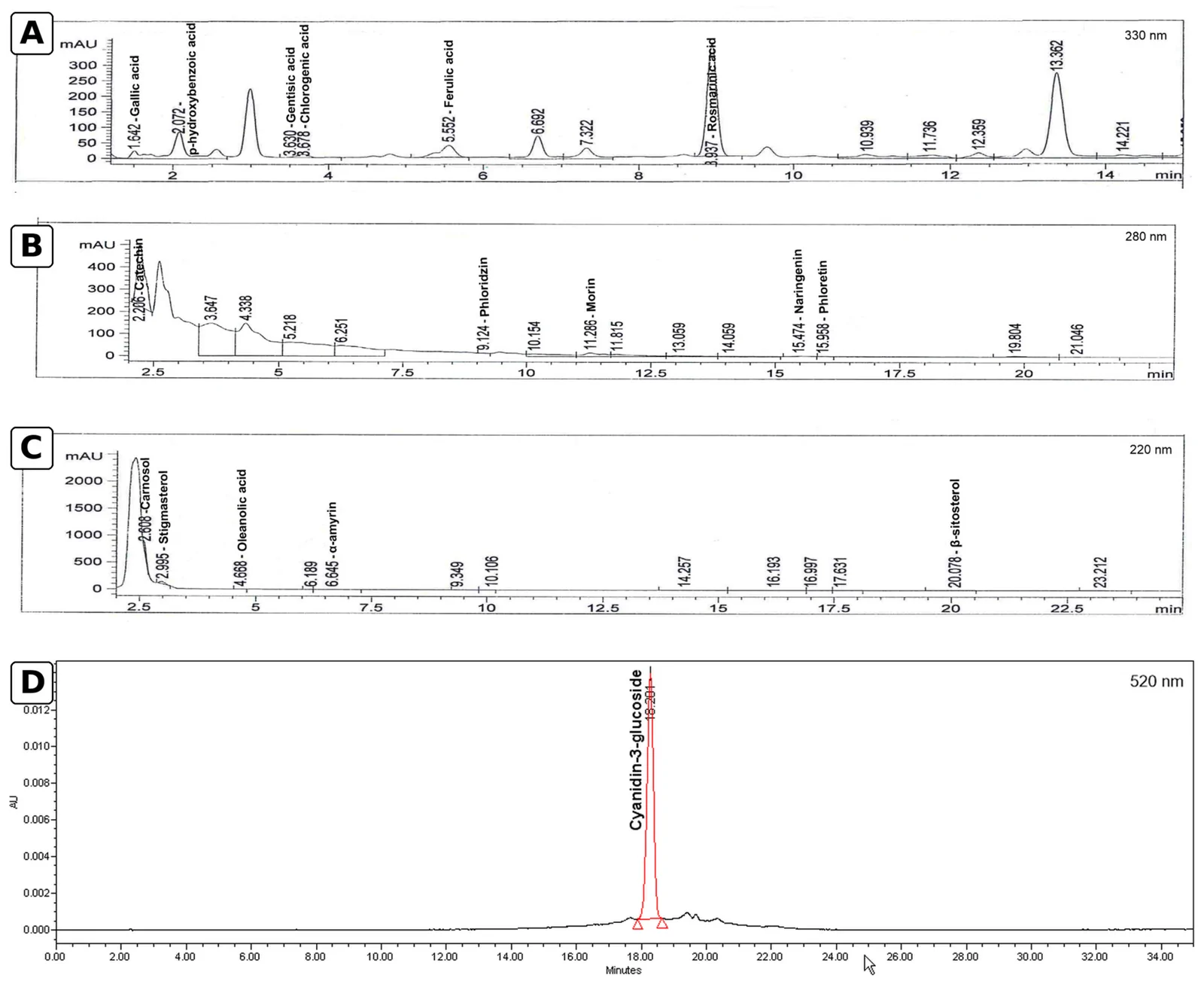
Representative HPLC chromatograms of the fresh flower aqueous extract of *C. pentadactylon*. (**A**) Phenolic acids detected at 330 nm. (**B**) Flavonoids detected at 280 nm. (**C**) Terpenoids and phytosterols detected at 220 nm. (**D**) Cyanidin 3-O-glucoside detected at 520 nm. Peak assignments were based on retention-time and UV–visible spectral concordance with authentic reference standards under the chromatographic conditions described in the Section 4.

The chromatographic profiles recorded at 330, 280, 220, and 520 nm supported the identification of phenolic acids, flavonoids, terpenoids/phytosterols, and cyanidin 3-O-glucoside, respectively (Figure 1). Compound assignments were based on the concordance between retention times and UV–visible absorption profiles and those of authentic reference standards analyzed under the same conditions. These assignments support the corresponding compounds but do not provide the level of structural confirmation achievable through mass spectrometry or nuclear magnetic resonance.

Integration of the literature-derived and experimentally detected constituents produced a final dataset of 36 unique compounds. Of these, 32 had previously been reported in the literature, 17 were detected in the FFAE, and both sources shared 13. Morin, gentisic acid, ursolic acid, and rosmarinic acid were detected in the experimental extract but were absent from the original 32-compound literature list (Table 1).

### 2.2. In Silico Toxicological Endpoint Predictions

The 36 compounds were evaluated across five organ- or effect-specific toxicological endpoints; predicted acute oral toxicity and reproductive-effects alerts were summarized separately. Across the complete non-redundant inventory of 36 compounds, hERG-related cardiotoxicity was the most frequent positive prediction (33/36; 91.7%), followed by hepatotoxicity (29/36; 80.6%), nephrotoxicity and carcinogenicity (28/36 each; 77.8%), and mutagenicity (21/36; 58.3%). These percentages represent compound-level screening frequencies and not the expected incidence of adverse effects in an exposed population. Predicted acute oral toxicity was evaluated for all 36 compounds (Table 2). Six compounds (16.7%) showed higher acute toxicity potential, defined operationally as a predicted LD_50_ of 1000 mg/kg or less; eight compounds (22.2%) showed intermediate potential (>1000–2000 mg/kg); and 22 compounds (61.1%) showed lower potential (>2000 mg/kg) (Table 2). Quercetin and myricetin had the lowest predicted LD_50_ values (159 mg/kg), followed by phloretin (500 mg/kg), stigmasterol and beta-sitosterol (890 mg/kg), and dacosanol beta-1 (1000 mg/kg).

### 2.3. Toxicological Alert Score and Compound Categorization

Using the prespecified normalized five-endpoint TAS thresholds, one compound had a lower relative alert burden (1/36; 2.8%), 12 had an intermediate burden (12/36; 33.3%), and 23 had a higher burden (23/36; 63.9%) (Table 3). These operational categories support prioritization and do not represent probabilities or demonstrated toxicity.

### 2.4. Compound–Toxicological Endpoint Network

**Figure 2:**
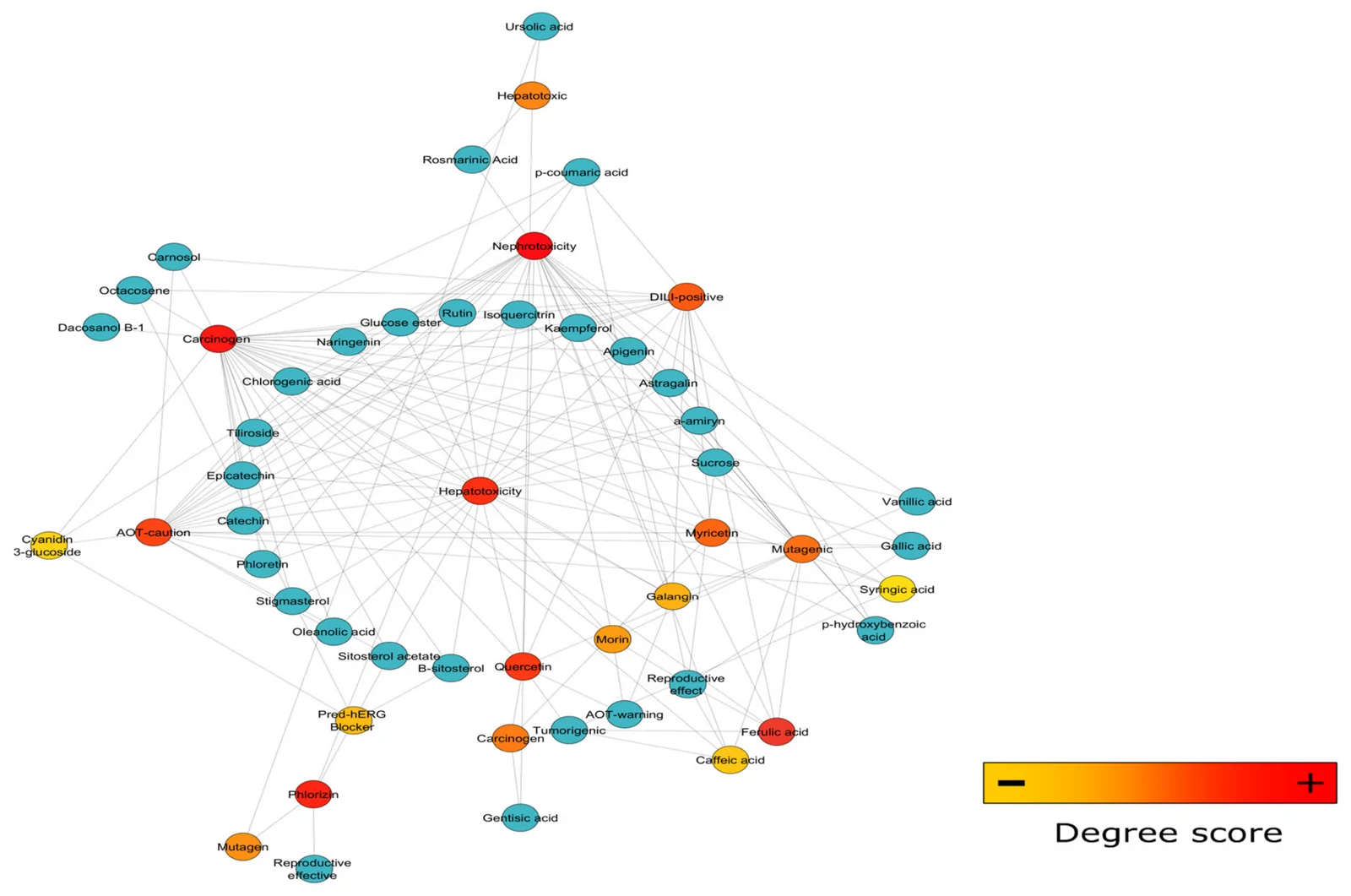
Primary compound–toxicological endpoint network for *C. pentadactylon*. Compound nodes are linked only to positive classifications for the five endpoints included in the TAS: hepatotoxicity, nephrotoxicity, mutagenicity, carcinogenicity, and hERG inhibition. Degree represents the number of connected positive endpoints or compounds. Reproductive-effects alerts and predicted LD_50_ classifications are excluded. Connectivity supports visualization and prioritization but does not constitute independent evidence of toxicity.

The primary bipartite network connected the evaluated phytochemicals with positive classifications for the five TAS endpoints (Figure 2). hERG inhibition was the most connected endpoint node (33 compounds), followed by hepatotoxicity (29), nephrotoxicity and carcinogenicity (28 each), and mutagenicity (21). Fourteen compounds were positive for all five endpoints: cyanidin 3-O-glucoside, tiliroside, astragalin, isoquercitrin, caffeic acid, ferulic acid, p-coumaric acid, rutin, phlorizin, myricetin, quercetin, apigenin, kaempferol, and galangin. Because the compound degree was identical to the number of positive TAS endpoints, the network was interpreted as a visualization of the alert matrix rather than independent toxicological evidence.

### 2.5. Integrative Toxicity Prediction Mode lITPM-Based Toxicological Assessment of the C. pentadactylon Flower Extract

**Figure 3:**
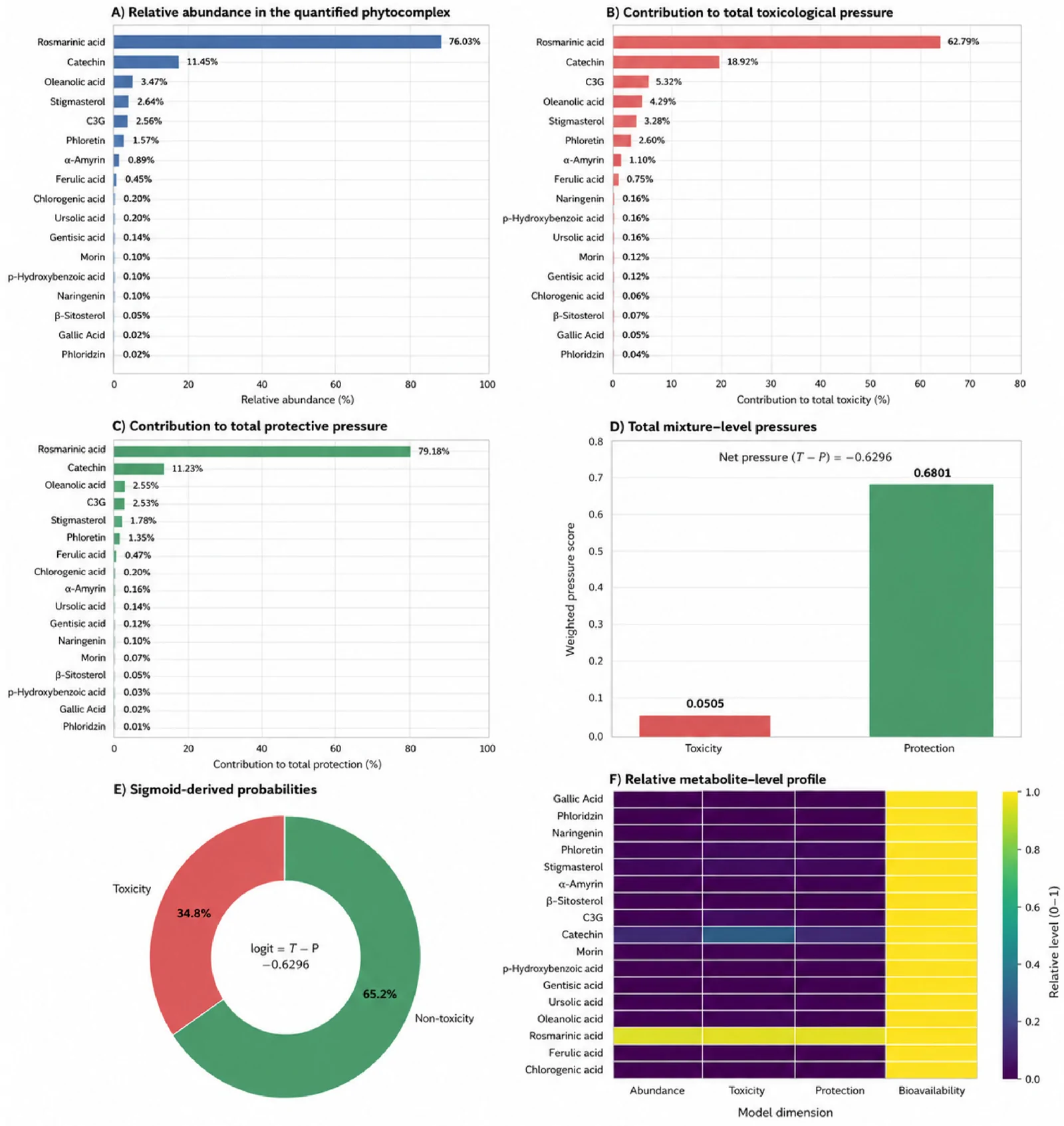
Integrative Toxicity Prediction Model (ITPM) applied to the 17 compounds quantified in the FFAE. (**A**) Relative abundance. (**B**) Individual toxicity-evidence contribution. (**C**) Individual protective evidence contribution. (**D**) Total toxicity- and protective evidence contributions. (**E**) Proportional toxicity- and protective evidence indices calculated directly using Equation (5). (**F**) Heatmap of abundance, toxicological evidence, protective evidence, and assigned bioavailability. No sigmoid transformation was applied. Outputs are comparative indices, not calibrated probabilities of toxicity or safety.

The ITPM was applied separately to the 17 compounds quantified in the FFAE and to the literature-informed dataset of 20 quantitatively reported compounds. For the 17-compound experimental profile, the total toxicity- and protective evidence contributions were 0.05045 and 0.68009, respectively. The resulting proportional toxicity-evidence index was 6.91%, with a complementary protective evidence proportion of 93.09% (Figure 3). No sigmoid transformation was applied because the proportional index is already bounded between 0 and 1 and no independently calibrated sigmoid parameters were available.

Due to its 76.03% share in the phytocomplex, rosmarinic acid accounted for most of the toxicity and protection, at 62.79% and 79.18%, respectively. With respect to toxicity, catechin represented 18.92%, and in terms of protection, it represented 11.23%. For the literature-informed 20-compound profile, total toxicity- and protective evidence contributions were 0.07096 and 0.29594, producing a proportional toxicity-evidence index of 19.34% and a protective evidence proportion of 80.66%. These model-derived values are comparative indices and do not demonstrate biological neutralization of toxicological liabilities.

### 2.6. Monte Carlo Uncertainty Analysis

**Figure 4:**
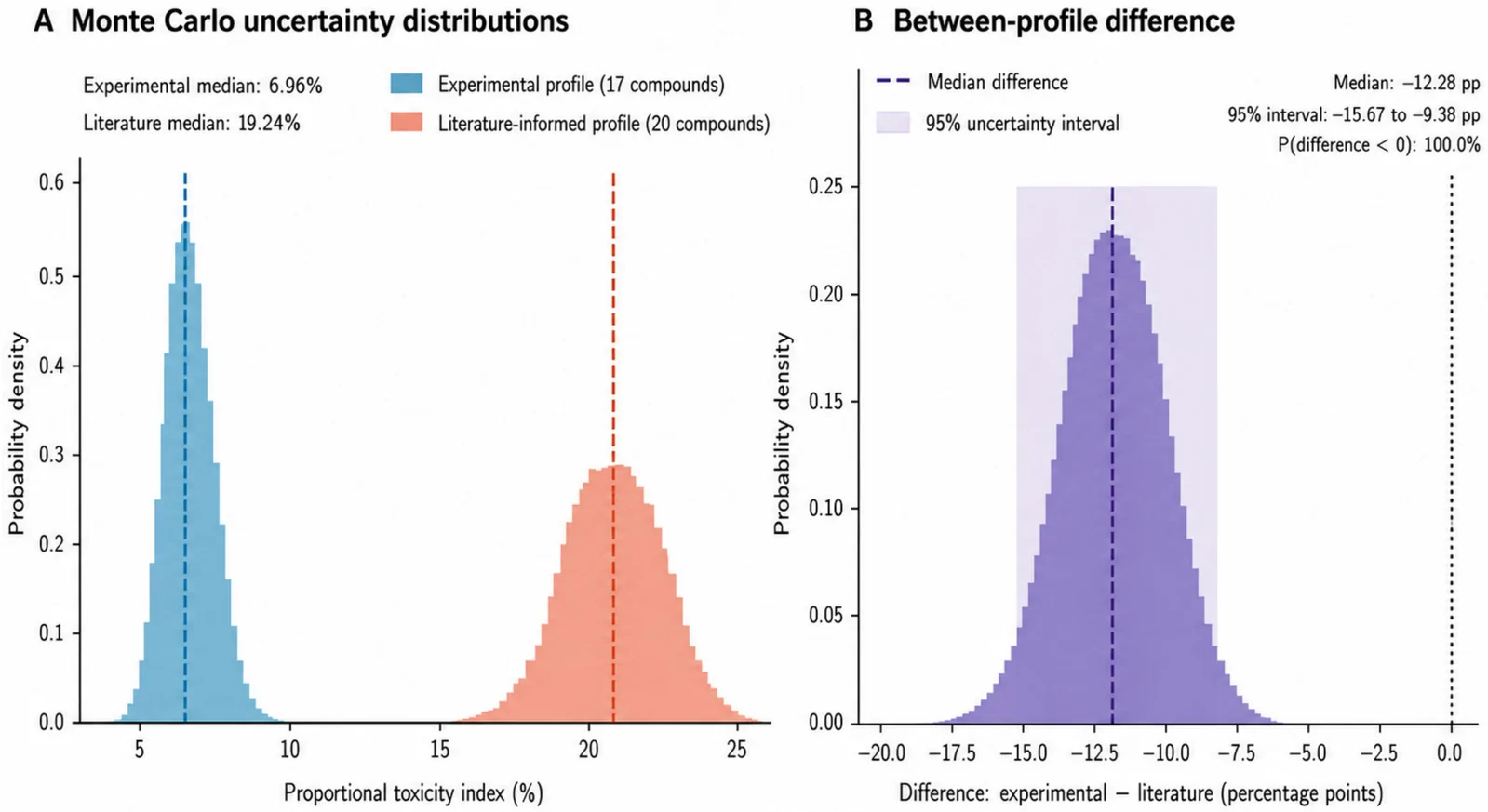
Monte Carlo uncertainty distributions for the proportional ITPM toxicity index of the 17-compound experimental FFAE profile and the 20-compound literature-informed profile. Distributions were generated from 50,000 iterations; vertical summaries show the central estimates and 95% uncertainty intervals. Simulated iterations represent propagated parameter uncertainty rather than biological replicates.

Uncertainty was propagated through 50,000 Monte Carlo iterations for the 17-compound experimental profile and the 20-compound literature-informed profile. The experimental profile produced a mean proportional toxicity index of 6.98%, a median of 6.96%, and a 95% uncertainty interval of 5.65–8.44%. The literature-informed profile produced a mean of 19.32%, a median of 19.24%, and a 95% uncertainty interval of 16.76–22.37%. The median difference was −12.28 percentage points (95% uncertainty interval: −15.67 to −9.38), and the experimental index was lower than the literature-informed index in 100% of the simulated iterations (Figure 4). Thus, the difference between the profiles remained robust under the evaluated uncertainty in compound concentrations and evidence scores.

A sensitivity analysis was conducted to determine the influence of alternative assumptions regarding the relative contribution of toxicological and protective evidence. Compared with the base-case median of 6.95%, the assumption favoring protective evidence reduced the median index to 4.29% (95% uncertainty interval: 3.47–5.22%), corresponding to a change of −2.66 percentage points. Conversely, the assumption favoring toxicological evidence increased the median to 11.07% (9.09–13.31%), representing a change of +4.12 percentage points. Although the numerical magnitude of the index was sensitive to these alternative assumptions, the experimental profile remained within the very-low predicted-concern interval.

Leave-one-compound-out analysis identified rosmarinic acid as the most influential constituent. Its exclusion increased the median toxicity index from 6.95% to 11.72% (10.17–13.45%), representing an increase of 4.77 percentage points. Removing catechin slightly decreased the median index to 6.38% (5.10–7.92%; −0.57 percentage points), whereas simultaneous exclusion of rosmarinic acid and catechin increased it to 12.40% (11.09–13.84%; +5.45 percentage points). Therefore, the qualitative classification of the experimental profile was preserved in every sensitivity and leave-one-compound-out scenario, although its numerical value was substantially influenced by the high abundance and protective evidence score assigned to rosmarinic acid. This influence should be interpreted as model dependence and not as evidence that rosmarinic acid biologically neutralizes the predicted toxic effects of the remaining constituents.

### 2.7. Preliminary External Evaluation of the ITPM

The standardized ITPM correctly classified 10 of 12 reference plant species, resulting in an overall accuracy of 83.3% and a balanced accuracy of 82.2%. Macro-averaged precision and recall were 0.822, and the macro-averaged F1 score was 0.815. Agreement with the literature-derived ordinal categories was substantial according to Cohen’s kappa (κ = 0.747) and high when category ordering was considered using quadratic-weighted kappa (κw = 0.890). The two discordant classifications occurred between adjacent categories, and no plant was misclassified directly between the low- and high-toxicity groups. These findings show preliminary discriminatory capacity but not definitive external validation.

### 2.8. Acute Oral Toxicity of the FFAE in Mice

No mortality, overt clinical signs of toxicity, or treatment-related behavioral abnormalities were observed after a single oral administration of FFAE at 300, 1000, 2000, or 5000 mg/kg during the 14-day observation period.

**Figure 5:**
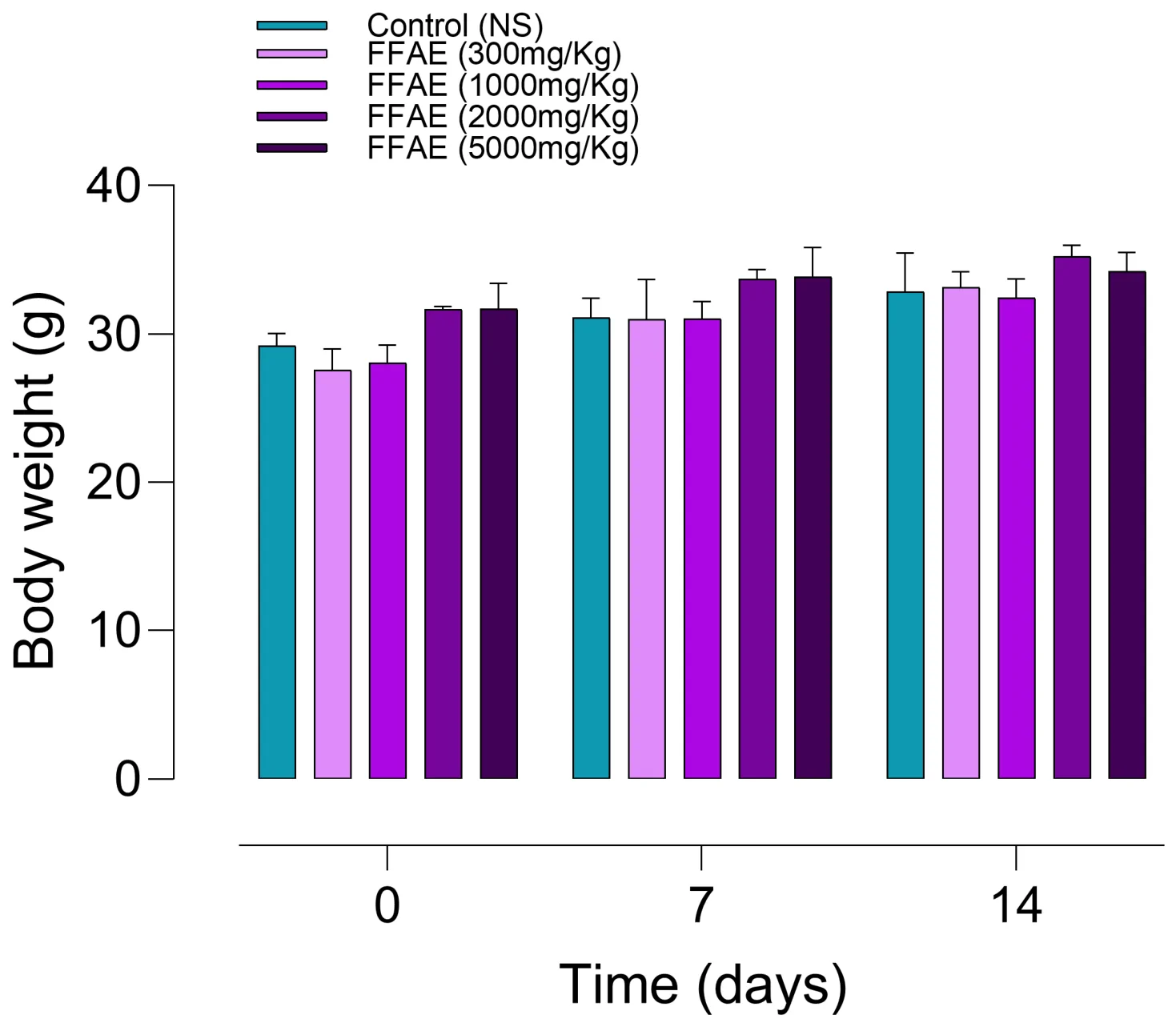
Body weight of male CD-1 mice following a single oral administration of the fresh flower aqueous extract of *C. pentadactylon*. Animals received saline (control) or FFAE at 300, 1000, 2000, or 5000 mg/kg, and body weight was recorded before administration and on days 7 and 14. Bars represent the mean ± standard error of the mean (SEM) (*n* = 5 animals per group). No statistically significant treatment-related differences were detected by two-way repeated-measures ANOVA followed by Dunnett’s multiple-comparison test (*p* > 0.05).

Body weight increased over time in the control and all FFAE-treated groups (Figure 5). Body weight in groups receiving 300 and 1000 mg/kg was similar to the control group. Those receiving 2000 and 5000 mg/kg also maintained or gained weight. Repeated-measures analysis did not identify significant treatment-related differences in body weight at the evaluated time points. A single FFAE administration produced no mortality, overt clinical abnormalities, or significant weight loss under the conditions tested, supporting low overt acute toxicity rather than general safety.

## 3. Discussion

This study integrated extract-specific phytochemical characterization, compound-level computational hazard screening, mixture-level evidence weighting, uncertainty analysis, and acute oral observations of the *C. pentadactylon* FFAE. This sequence is important because a hazard alert assigned to an isolated molecule is not equivalent to the risk posed by a phytocomplex. Risk interpretation also depends on the amount of each constituent in the administered material, its bioavailability and metabolism, interactions among constituents, and the exposure regimen. Accordingly, the computational outputs reported here should be regarded as screening and prioritization evidence rather than as direct probabilities of adverse effects in vivo [14,40,41,42,43,44,45].

The analysis found 17 metabolites in the extract, totaling 132.73 mg/g. Rosmarinic acid predominated (100.91 mg/g; 76.03% of the quantified fraction), followed by catechin (15.20 mg/g; 11.45%), oleanolic acid (4.60 mg/g; 3.47%), stigmasterol (3.51 mg/g; 2.64%), cyanidin-3-O-glucoside (3.42 mg/g; 2.58%), phloretin (2.09 mg/g; 1.57%), and alpha-amyrin (1.18 mg/g; 0.89%). Thus, the modeled composition was driven mainly by two phenolic constituents rather than by an equal contribution from all detected compounds. The 17 quantified metabolites overlapped partly with the 32 compounds reported for the species in the literature, producing a nonredundant inventory of 36 compounds. This difference between the literature inventory and the aqueous-extract profile is chemically plausible because solvent polarity, plant organ, origin, processing, and analytical conditions influence the recovered phytochemical spectrum [28,38,39]. It also supports using measurements from the exact test article when estimating mixture-level behavior.

At the compound level, the most frequent positive predictions were hERG-related cardiotoxicity alerts (33/36; 91.7%), hepatotoxicity (29/36; 80.6%), nephrotoxicity (28/36; 77.8%), and carcinogenicity (28/36; 77.8%); mutagenicity was predicted for 21/36 compounds (58.3%), and supplementary reproductive-effects alerts were identified for 7/36 (19.4%). These frequencies describe the number of compounds flagged by the selected prediction rules and do not represent the incidence of toxicity in animals or humans. An hERG alert addresses a defined electrophysiological liability and should not be interpreted as a comprehensive cardiotoxicity diagnosis. Positive predictions for hepatic, renal, mutagenic, or carcinogenic endpoints require confirmation in appropriate experimental systems, whereas the reproductive-effects outputs should be regarded as supplementary structural alerts requiring endpoint-specific evaluation. Thirty compounds (83.3%) had predicted LD_50_ values above 1000 mg/kg; however, only 22 (61.1%) were above 2000 mg/kg. These classifications describe predicted acute-lethality potential and do not exclude organ-specific or repeated-dose toxicity.

The normalized five-endpoint TAS placed 1/36 compounds (2.8%) in the lower, 12/36 (33.3%) in the intermediate, and 23/36 (63.9%) in the higher relative-alert category. The higher category therefore identifies compounds for closer evaluation and does not indicate demonstrated toxicity in the extract. Structural alerts such as catechol, Michael acceptor, hydroquinone, charged oxygen/sulfur, and isolated alkene can indicate plausible reactive motifs, but their biological expression depends on dose, accessibility, biotransformation, detoxification, and cellular context [46,47,48,49,50,51,52,53,54,55,56,57,58].

Similarly, the endpoint compound network is useful for visualizing shared alerts and highly connected nodes, although node degree is partly a mathematical restatement of the number of positive endpoints and should not be treated as independent mechanistic validation.

The primary network showed that 14 compounds were connected to all five TAS endpoints. This maximum degree reflects convergence of model alerts rather than toxic potency. Quercetin and myricetin warrant particular attention because both combined five positive endpoint classifications with comparatively low predicted LD_50_ values (159 mg/kg). Conversely, several highly connected compounds had higher predicted LD_50_ values, illustrating that organ- or mechanism-specific alerts and acute-lethality estimates convey different information. Confirmatory prioritization should therefore consider endpoint convergence together with abundance, bioavailability, metabolism, and exposure within the extract.

After abundance, toxicological evidence, protective evidence, and estimated bioavailability were integrated, the 17-compound experimental profile produced total toxicity- and protective evidence contributions of 0.05045 and 0.68009, respectively. These values corresponded to a proportional toxicity-evidence index of 6.91% and a protective evidence proportion of 93.09%. No sigmoid transformation was retained because Equation (5) already provides a bounded index and the parameters required for a nonlinear transformation had not been independently calibrated. These quantities are comparative model-derived indices and not probabilities of toxicity, protection, or safety.

Rosmarinic acid accounted for 62.79% of the modeled toxicity-evidence contribution and 79.18% of the protective evidence contribution, whereas catechin accounted for 18.92% and 11.23%, respectively. Their influence primarily reflected their abundance in the quantified fraction. Therefore, the predominance of rosmarinic acid is an expected consequence of abundance weighting rather than evidence that this compound determines the biological toxicity of the complete extract. The simultaneous assignment of toxicological and protective evidence is not inherently contradictory, because polyphenols may participate in antioxidant or cytoprotective pathways while exhibiting concentration-, redox-, or context-dependent liabilities [56,57,58]. Nevertheless, the predominance of modeled protective evidence does not demonstrate biological neutralization of toxicological liabilities or establish extract safety. The comparison with the 20-compound literature-informed profile shows why composition weighting materially changes interpretation. The latter produced a higher toxicity proportion (19.34%) than the extract-specific profile (6.91%), while its protective evidence proportion was lower (80.66% versus 93.09%). This contrast is consistent with the different abundance structures: the experimental profile was dominated by rosmarinic acid and catechin, whereas the literature-informed profile assigned substantial weight to other constituents, including alpha-amyrin. Therefore, the 17-versus-20 comparison should not be interpreted as an intrinsic effect of the number of compounds. This study examines two separate chemical scenarios: one from the extract tested and another compiled from published data, potentially reflecting variations in samples and how they were processed.

Monte Carlo propagation supported the numerical stability of this difference under the parameter distributions specified in the model. Across 50,000 iterations, the experimental profile had a mean toxicity proportion of 6.98% (95% uncertainty interval, 5.65–8.44%), whereas the literature-informed profile had a mean of 19.32% (16.76–22.37%). The median paired difference was −12.28 percentage points (95% uncertainty interval, −15.67 to −9.38), and the experimental profile was lower in all simulated iterations. The sensitivity analysis at B = 1 yielded a similar median difference (−12.27 percentage points). These intervals quantify uncertainty in the selected inputs and distributional assumptions; they do not encompass all biological variability, model-form uncertainty, unidentified constituents, or differences among extract batches. Consequently, the Monte Carlo analysis supports robustness within the operational model, not universal toxicological certainty.

The preliminary transportability analysis classified 10 of the 12 reference plant species consistently with their prespecified literature-based categories, corresponding to an overall agreement of 83.3%, a balanced accuracy of 82.2%, a macro-F1 score of 0.815, and a Cohen’s kappa of 0.747. The two discordant cases involved adjacent categories, suggesting potentially useful ordinal discrimination within this limited reference set. However, the small and chemically heterogeneous sample precludes definitive validation, and performance may change when species with different phytochemical profiles or toxicity mechanisms are examined. Larger, prospectively defined datasets, transparent reference-category assignment, applicability-domain analysis, and comparison with independent experimental outcomes are required before the framework can be considered externally validated [44,45,59,60].

The acute oral study provided an independent whole-animal layer of evidence. No mortality, overt clinical signs, or observable behavioral abnormalities occurred in male CD-1 mice following a single FFAE administration of 300, 1000, 2000, or 5000 mg/kg and 14 days of observation. Body weight increased over time in all groups, and no statistically significant treatment-related differences were detected relative to the control group using two-way repeated-measures ANOVA followed by Dunnett’s multiple-comparison test. These findings support low overt acute toxicity under the specific conditions evaluated. The comparatively low extract-specific toxicity-evidence index was directionally concordant with this observation; however, the computational and experimental findings were generated at different levels of analysis and do not establish a causal relationship or validate individual endpoint predictions. Moreover, the acute study does not exclude biochemical, histological, electrophysiological, genotoxic, reproductive, or repeated-dose effects that were not evaluated.

Several limitations define the scope of the conclusions. The animal study used five males per group, a single administration, and a 14-day observation period; it did not include females, organ weights, serum clinical chemistry, urinalysis, electrocardiography, gross pathology, or histopathology. Systemic exposure to the quantified parent compounds and their metabolites was not measured. The chemical model covered the 17 quantified metabolites, but unidentified or unquantified constituents and batch-to-batch variability remain possible. The computational endpoints were generated from heterogeneous models with distinct applicability domains, and the protective scores summarize literature-supported mechanisms rather than direct activity measurements in this extract. The ordinal evidence weights and consistency factors were operational modeling parameters and have not been empirically calibrated against quantitative toxicological outcomes. Monte Carlo propagation did not capture uncertainty in domain selection, possible model misclassification, unidentified constituents, or interactions among metabolites. Interactions among metabolites were not experimentally demonstrated [17,18,19,61,62,63,64,65,66].

Future work should therefore combine validated quantitative LC-MS/MS with replicate-batch analysis and toxicokinetic measurement of major constituents and metabolites. A repeated-dose study should include both sexes, food consumption, hematology, liver and kidney function markers, urinalysis, organ weights, and histopathology. Given the frequency of hERG-related alerts, electrocardiographic assessment and targeted ion-channel testing would be particularly informative; genotoxicity and reproductive testing should be added when supported by exposure and intended use. These data would allow recalibration of the ITPM against observed outcomes and would determine whether the present low acute-risk signal persists under repeated or clinically relevant exposure conditions.

## 4. Materials and Methods

### 4.1. Plant Material

Fresh flowers of *Chiranthodendron pentadactylon* Larreat were collected in April 2026 from the source tree CHPE1 in Metepec, State of Mexico, Mexico (19°15′48″ N, 99°37′19.6″ W). The tree belongs to a Management Unit for Wildlife Conservation (Unidad de Manejo para la Conservación de la Vida Silvestre, UMA; SEMARNAT-UMA-IN-325-MEX/21), and collection was authorized by its owner and legal representative, David Guajardo Ruz (Figure S1). The species is listed as threatened under NOM-059-SEMARNAT-2010 [67]. Botanical identification was performed by Dr. Jaime Jiménez Ramírez, and a voucher specimen was deposited in the Herbarium of the Faculty of Sciences, National Autonomous University of Mexico (UNAM; voucher 181307; Figure S2). Taxonomic nomenclature was verified using Enciclovida (https://enciclovida.mx/especies/172327-chiranthodendron-pentadactylon; accessed 8 August 2026).

### 4.2. Preparation of the Fresh Flower Aqueous Extract

Fresh flowers (135 g) were boiled in 1 L of double-distilled water for 15 min from the onset of boiling in a covered vessel. After cooling, the preparation was filtered through Whatman Grade 3 paper and dried under controlled airflow at 25 °C for 24 h. The resulting fresh flower aqueous extract (FFAE; 6.0 g; yield, 4.45%) was stored in an airtight, light-protected container at 4 °C for no longer than 2 months before analysis.

### 4.3. Chemicals and Reference Standards

HPLC-grade methanol and acetonitrile were obtained from LiChrosolv^®^ (Merck KGaA, Darmstadt, Germany) and J.T.Baker™ (Phillipsburg, NJ, USA), respectively. Trifluoroacetic acid, formic acid, and reference standards were obtained from Sigma-Aldrich (St. Louis, MO, USA). Ultrapure water was produced using a Milli-Q^®^ system(MilliporeSigma, Burlington, MA, USA). Mobile phases were filtered through 0.45 µm nylon membranes and degassed using an in-line degasser. Reference standards included phenolic acids (protocatechuic, p-hydroxybenzoic, vanillic, caffeic, β-resorcylic (α-resorcílico), 3,5-dihydroxybenzoic, gallic, syringic, p-coumaric, chlorogenic, sinapic, ferulic, and rosmarinic acids); flavonoids (rutin, morin, quercetin, catechin, hesperidin, phloridzin, phloretin, apigenin, myricetin, kaempferol, and isorhamnetin); cyanidin 3-O-glucoside chloride (kuromanin chloride); and terpenoids or phytosterols (carnosol, ursolic acid, α-amyrin, stigmasterol, oleanolic acid, α-myrcene, and β-sitosterol).

### 4.4. HPLC Characterization of the FFAE

Phenolic acids, flavonoids, terpenoids, and phytosterols were analyzed using an Agilent Technologies 1100 HPLC system (Agilent Technologies, Santa Clara, CA, USA) equipped with a G1312A binary pump, G1379A degasser, G1313 auto sampler and diode-array detector, and ChemStation software B.02.0x. The FFAE was dissolved in water–methanol (85:15, *v*/*v*) at 25 mg/mL, sonicated for 5 min, and filtered through a 0.45 µm nylon membrane.

#### 4.4.1. Phenolic Acids and Flavonoids

Separation was performed on a Hypersil ODS column (125 × 4.0 mm, 5 µm; Thermo Fisher Scientific, Bellefonte, PA, USA). The mobile phase comprised water adjusted to pH 2.5 with trifluoroacetic acid (A) and acetonitrile (B). The gradient was 15% B at 0–0.1 min, 15–35% B at 0.1–20 min, and 35% B at 20–25 min, followed by 5 min of re-equilibration. The flow rate was 1.0 mL/min, injection volume 20 µL, and column temperature 30 °C. Detection was performed at 254, 280, 330, and 365 nm. Compounds were assigned by comparison with the retention times and UV spectra of authentic standards [55].

#### 4.4.2. Terpenoids and Phytosterols

Separation was performed on a ZORBAX Eclipse XDB-C8 column (125 × 4.0 mm, 5 µm; Agilent Technologies, Santa Clara, CA, USA) using isocratic acetonitrile–water (80:20, *v*/*v*) at 1.0 mL/min. The injection volume was 20 µL, the column temperature was 40 °C, and the detection wavelengths were 215 and 220 nm. The run time was 25 min, followed by 5 min of column washing and re-equilibration.

#### 4.4.3. Cyanidin 3-O-Glucoside

Cyanidin 3-O-glucoside was analyzed using a Waters HPLC system (Waters Corporation, Milford, MA, USA), which comprising a model 600 pump controller, a 717 Plus autosampler, and a 2487 dual-wavelength UV/Vis detector. Separation was performed on a ZORBAX SB-C18 column (4.6 × 150 mm, 5 µm) using water containing 1% formic acid (A) and methanol containing 1% formic acid (B). The gradient was 15% B at 0–2 min, 15–45% B at 2–32 min, 45–15% B at 32–33 min, and 15% B at 33–40 min. The flow rate was 0.8 mL/min, the injection volume was 10 µL, the column temperature was 30 °C and the detection wavelength was 520 nm. Kuromanin chloride was used as the reference standard.

### 4.5. In Silico Toxicological Assessment

#### 4.5.1. Phytochemical Dataset

PubMed and Scopus were searched through 30 April 2026 using database-adapted combinations of (“*Chiranthodendron pentadactylon*” OR “flor de manita” OR “hand flower tree” OR “devil’s hand flower” OR “macpalxochitl”) AND (compound\OR phytochemical\OR constituent\OR metabolite\). Studies identifying compounds specifically in the flowers through chromatographic, spectroscopic, or spectrometric techniques were included. Compounds detected in the FFAE were added, and duplicates were removed.

The final dataset comprised 36 unique compounds: 32 reported in the literature and 17 detected in the FFAE, with 13 compounds shared by both sources and four exclusive to the experimental extract. Compound names, chemical classes, PubChem Compound Identifiers, origins, and references are provided in Supplementary Table S3. Canonical SMILES and SDF structures were obtained from PubChem; when required, SMILES were converted to SDF using Open Babel 3.2.0. The evaluated endpoints were hepatotoxicity, nephrotoxicity, Ames mutagenicity, carcinogenicity, acute oral toxicity, and hERG channel inhibition. Model details and interpretation rules are summarized in Supplementary Table S2.

#### 4.5.2. Toxicological Endpoint Predictions

Hepatotoxicity was predicted using ProTox 3.0, DL-DILI, and the IRFMN hepatotoxicity model v1.0.1 implemented in VEGA 1.1.5. DL-DILI outputs were interpreted specifically as predictions of drug-induced liver injury. Nephrotoxicity was assessed using the organ-toxicity model implemented in ProTox 3.0 and was reported as a single-model prediction.

Ames mutagenicity was evaluated using ProTox 3.0, the Benigni–Bossa rule base in Toxtree 3.0, DataWarrior 6.1.0, and the CAESAR Ames model implemented in VEGA 1.1.5. Carcinogenicity was assessed using ProTox 3.0, the CAESAR carcinogenicity model implemented in VEGA 1.1.5, and the Benigni–Bossa rule base in Toxtree 3.0.

Potential hERG channel inhibition was evaluated using Pred-hERG version 4.2. A compound was considered positive for hERG-related liability only when the final weighted-ensemble classification was “blocker”; “non-blocker” predictions were considered negative, whereas predictions outside the relevant applicability domain were recorded as not evaluable and did not constitute positive votes. Regression and multiclass outputs were retained as supporting information. A predicted pIC_50_ ≥ 4.5, corresponding to an IC_50_ ≤ 31.6 µM, indicated at least weak hERG-blocking potential. Potency was classified as weak at pIC_50_ 4.5–<5.0, moderate at 5.0–<6.0, and strong at ≥6.0, whereas values < 4.5 were classified as non-blocking. hERG positivity was interpreted as a potential channel-blocking or arrhythmogenic alert rather than evidence of comprehensive cardiotoxicity. Acute oral toxicity was evaluated using ProTox-III, DL-AOT, and BESTox. The predicted LD_50_ values reported in Table 4 correspond to the point estimates generated by ProTox-III and do not represent the mean or median of the three servers. DL-AOT and BESTox outputs were used as independent supporting predictions for endpoint-level consensus classification. A positive acute-toxicity alert was assigned when at least two evaluable servers supported an LD_50_ ≤ 2000 mg/kg or the corresponding server-specific acute-toxicity category. Predictions above 2000 mg/kg were considered negative for this binary endpoint, whereas unavailable or non-evaluable outputs were retained as missing information and were not counted as negative predictions. Supplementary Table S4 presents the original server-level acute oral toxicity outputs without averaging across platforms. For each compound, it includes the ProTox-III LD_50_ estimate in mg/kg, predicted toxicity class and probability; the original DL-AOT regression value in log_10_(mg/kg), its back-transformed LD_50_ estimate, multiclass label and associated probability; and the BESTox LD_50_ estimate in mg/kg. Applicability-domain or reliability information, when provided by the corresponding platform, is reported separately, together with the final binary consensus and the numbers of evaluable and positive servers.

#### 4.5.3. Consensus, Uncertainty, and Supplementary Alerts

Outputs were standardized as positive, negative, inconclusive, or not evaluable (NE). For endpoints assessed using multiple independent tools, a positive classification required at least two positive predictions; a negative classification required at least two negative predictions and no positive prediction. Discordant results were inconclusive, whereas endpoints with fewer than two evaluable predictions were NE. Predictions outside the applicability domain or with insufficient reliability were classified as NE when the relevant information was available. Single-model nephrotoxicity and hERG predictions were not described as consensus classifications.

DataWarrior 6.1.0 reproductive-effects outputs were treated as supplementary evidence and excluded from the primary Toxicological Alert Score (TAS). Brenk alerts identified using SwissADME (accessed 14 March 2026) were treated as medicinal-chemistry alerts. Structural similarity was evaluated against a toxicologically relevant reference set from the Therapeutic Target Database (2026 release; accessed 17 April 2026) using Morgan fingerprints of radius 2 and the Tanimoto coefficient. Similarity ≥ 0.70 was considered structurally related but did not determine toxicological classification.

### 4.6. Toxicological Alert Score

The exploratory TAS summarized positive classifications across five organ- or effect-specific endpoints: hepatotoxicity, nephrotoxicity, Ames mutagenicity, carcinogenicity, and hERG inhibition. One point was assigned per positive endpoint, and the normalized TAS was calculated as the number of positive endpoints divided by five. Predicted acute oral toxicity was reported separately as an LD_50_-based classification because it represents an acute-lethality estimate rather than an additional organ- or mechanism-specific alert in the primary matrix. Reproductive-effects, Brenk, Cramer, PAINS, general structural alerts, and similarity findings did not contribute to the primary TAS. Inconclusive and NE results received no point but were retained as uncertainty indicators. Normalized values were categorized operationally as lower (<0.33), intermediate (0.33 to <0.67), or higher (≥0.67) relative alert burden. These categories are exploratory prioritization bands, not probabilities of toxicity, clinical-risk levels, or evidence of extract safety.

### 4.7. Toxicological Endpoint Connectivity of C. pentadactylon Phytochemicals

A bipartite network was generated using Cytoscape 3.10.4 and cytoHubba [68,69]. Edges represented positive classifications for the five endpoints included in the TAS; predicted LD_50_ and supplementary alerts were excluded from the primary network. Degree represented the number of positive endpoints per compound or implicated compounds per endpoint. Compounds with the maximum degree and the two highest-degree endpoints were identified for exploratory prioritization. Because compound degree was directly related to TAS, the network was used for visualization and not as independent toxicological evidence.

### 4.8. Integrative Toxicity Prediction Model

#### 4.8.1. Compound-Level Scoring and Mixture Integration

The Integrative Toxicity Prediction Model (ITPM) integrated relative phytochemical abundance, estimated bioavailability, toxicological evidence, and reported protective activities. Relative abundance was calculated as:

(1) $$A_{i}=\frac{C_{i}}{\sum_{j=1}^{n}C_{j}}$$

where (*C_i_*) is the concentration of compound (*i*), (*n*) is the number of compounds included in the corresponding scenario, and (*A_i_*) is its relative abundance.

Toxicological evidence was evaluated across six domains: hepatotoxicity, nephrotoxicity, genotoxicity/mutagenicity, carcinogenicity, cardiotoxicity, and reproductive toxicity. The highest eligible level of positive evidence in each domain was scored as 0.25 (in silico), 0.50 (in vitro), 0.75 (in vivo), or 1.00 (human). The Multidomain Toxicological Evidence Score (MTES) was calculated as:

(2) $${MTES}_{i}=\left(\frac{\sum_{d=1}^{D_{i}}{TE}_{i.d}}{D_{i}}\right)\left(\frac{D_{i}}{6}\right){TR}_{i}$$

where (*TE[68]*) is the toxicological evidence score for compound (*i*) in domain (*d*), and (*TR_i_*) is the consistency factor. The latter was set to 1.00 for concordant evidence from at least two independent sources, 0.75 for evidence supported by one direct experimental study, or 0.50 for predominantly computational, limited, or inconsistent evidence.

Protective evidence was evaluated independently across antioxidant, anti-inflammatory, cytoprotective, antimutagenic, antigenotoxic, and xenobiotic-defense domains. Evidence was scored as 0.50 (in vitro), 0.75 (in vivo), or 1.00 (human). The Multidomain Protective Evidence Score (MPES) was calculated as:

(3) $${MPES}_{i}=\left(\frac{\sum_{d=1}^{D_{i}}E_{i.d}}{D_{i}}\right)\left(\frac{D_{i}}{6}\right)R_{i}$$

where (*E[68]*) is the protective evidence score for compound (*i*) in domain (*d*), and (*R_i_*) is the corresponding consistency factor. This factor was set to 1.00 for concordant evidence from at least two independent studies, 0.75 for one direct study, or 0.50 for limited, inconsistent, mixture-derived, or structural-analog evidence. Domains without eligible positive evidence received no contribution and were recorded as not documented rather than as evidence of absence.

The MTES and MPES contributions of each compound were weighted by relative abundance and estimated bioavailability:

(4) $$T_{\omega ,i}=A_{i}B_{i}{MTES}_{i};\;P_{\omega ,i}=A_{i}B_{i}{MPES}_{i}$$

where (*B_i_*) is the estimated bioavailability of compound (i), whereas (*T_ω,i_*) and (*P_ω,i_*) represent its weighted toxicological and protective evidence contributions, respectively.

The compound-level contributions were summed separately to obtain the total toxicological and protective evidence components:

(5) $$T_{total}=\sum_{i}T_{\omega ,i};\;P_{total}=\sum_{i}P_{\omega ,i}$$

Their proportional representation within each phytochemical scenario was calculated as:

(6) $${ITPM}_{toxicity}=\frac{T_{total}}{T_{total}+P_{total}};\;{ITPM}_{non-toxicity}=1-{ITPM}_{toxicity}$$

Equation (6) was applied only when:

(7) $$T_{\mathrm{total}}+P_{\mathrm{total}}>0$$

Otherwise, the proportional indices were considered not estimable.

The evidence weights and consistency factors were predefined as ordinal scaling parameters rather than empirically calibrated effect sizes. Toxicological and protective evidence were modeled as parallel components; therefore, protective evidence did not imply prevention, compensation, or neutralization of toxicological liabilities. Because the proportional indices were already bounded between 0 and 1, the previous sigmoid transformation was not retained. The resulting outputs represent comparative evidence-weighted indices rather than probabilities of toxicity or protection, clinical-risk estimates, or experimentally demonstrated biological interactions. Detailed scoring criteria and compound-level assignments are provided in the Supplementary Methods.

#### 4.8.2. Uncertainty and Sensitivity Analyses

The ITPM was applied to an extract-specific scenario comprising 17 compounds quantified in the experimentally tested FFAE and a literature-informed scenario comprising 20 quantitatively reported constituents (Supplementary Table S3). The latter represented a broader species-level profile and was not assumed to reproduce the experimental extract.

Uncertainty was propagated through 50,000 Monte Carlo iterations per scenario. Concentrations were sampled from log-normal distributions centered on the reported values (CV, 20%); MTES and MPES from bounded distributions centered on their nominal values (CV, 10%; range, 0–1); and bioavailability from triangular distributions centered on the assigned values and extending ±15% (range, 0–1).

For morin, gentisic acid, and ursolic acid, which lacked directly comparable six-domain computational profiles, MTES was sampled uniformly between 0 and 0.125. The upper limit corresponds to the maximum score attainable when all six domains contain computational-only positive evidence and the predominantly computational consistency factor is applied: 0.125.

Relative abundances, weighted contributions, total evidence components, and proportional ITPM indices were recalculated at each iteration. The distributions were summarized using the mean, median, standard deviation, interquartile range, and 95% uncertainty interval.

The between-scenario difference was calculated at each paired iteration as:

(8) $$∆ITPM={ITPM}_{extract}-{ITPM}_{literature}$$

$${ITPM}_{toxicity-evidence}=\frac{T_{total}}{\left(T_{total}+P_{total}\right)};{ITPM}_{protective-evidence}=\frac{T_{total}}{\left(T_{total}+P_{total}\right)}=1-{ITPM}_{toxicity-evidence}.$$

(9) $${ITPM}_{toxicity-evidence}=\frac{T_{total}}{T_{total}+P_{total}}$$

(10) $${ITPM}_{protective-evidence}=\frac{P_{total}}{T_{total}+P_{total}}=1-{ITPM}_{toxicity-evidence}$$

where:

*T_total_* = total toxicological pressure score obtained by summing the weighted toxicological contributions of the evaluated metabolites.
*P_total_* = total protective pressure score obtained by summing the weighted protective contributions of the evaluated metabolites.
*ITPM_toxicity-evidence_* = proportional toxicological evidence within the total integrated toxicity–protection balance; values range from 0 to 1.
*ITPM_protective-evidence_* = proportional protective evidence within the total integrated toxicity–protection balance; values range from 0 to 1 and are complementary to the toxicity-evidence index.
Interpretation note. These indices represent the relative mathematical balance of toxicological and protective evidence integrated by the ITPM; they should not be interpreted as experimentally calibrated probabilities of toxicity, non-toxicity, or safety.

#### 4.8.3. Preliminary External Evaluation

The ITPM underwent a preliminary proof-of-concept evaluation of model transportability using 12 species assigned literature-informed ordinal reference categories of low, moderate, or high toxicological concern. Reference categories were assigned independently of the calculated ITPM outputs using a predefined evidence hierarchy that prioritized regulatory and governmental assessments, followed by systematic reviews and peer-reviewed clinical toxicology reports. Low concern denoted species with authoritative support for traditional use under specified conditions and without a predominant severe characteristic hazard; moderate concern indicated credible but preparation-, dose-, duration-, route-, or susceptibility-dependent serious toxicity; and high concern denoted well-established severe, potentially fatal, carcinogenic, nephrotoxic, neurotoxic, or cardiotoxic hazards associated with the plant or its defining constituents. These categories were treated as literature-informed comparative labels rather than regulatory classifications or observed clinical outcomes. The purpose was to determine whether the predefined framework broadly preserved these ordinal categories when applied without retraining, recalibration, species-specific multipliers, or post hoc adjustment; it was not intended as definitive external validation. The same equations, weights, and thresholds were applied uniformly, and model outputs were categorized as low (<40%), moderate (40–<60%), or high (≥60%). Performance was evaluated using a confusion matrix, overall and balanced accuracy, macro-averaged precision, recall and F1 score, Cohen’s kappa, and quadratic-weighted kappa. Two adjacent-category discordances were identified: *Piper methysticum* (Kava) was classified as moderate in the literature-informed reference set but predicted as low by the ITPM (38.48%), whereas *Dysphania ambrosioides* was assigned a high reference category but predicted as moderate (59.48%). Both predictions were close to their respective 40% and 60% decision boundaries. Because the reference set was small and chemically and preparation-wise heterogeneous, performance metrics were interpreted descriptively and as hypothesis-generating. The species-level assignment rules, supporting references, taxonomic and preparation-related limitations, and calculations are provided in the Supplementary Methods and Tables.

### 4.9. Acute Oral Toxicity Study

#### 4.9.1. Animals and Ethical Approval

Twenty-five 5-week-old male CD-1 mice weighing 25–30 g were housed in transparent acrylic cages at 21–23 °C under a 12 h light/dark cycle, with 5001 Rodent Laboratory Chow and water ad libitum. Animals were acclimatized for seven days and housed at five mice per cage under 40–60% relative humidity. Animal procedures followed NOM-062-ZOO-1999 and the 2011 edition of the Guide for the Care and Use of Laboratory Animals. Biological waste was handled according to NOM-087-SEMARNAT-SSA1-2002. The study was approved by three committees. First, the Research Committee assessed the project’s technical quality, scientific value, feasibility, and coherence. Second, the Ethics committee reviewed risks and benefits, upholding human rights and informed consent. Third, the CICUAL (Committee for the Care and Use of Laboratory Animals) focused on research ethics and the care of animals within the Faculty of Medicine at UNAM. Our protocol, FM/DI/029/2026, and CICUAL approval number 010/CICUAL were granted approval on 5 May 2026.

#### 4.9.2. Experimental Procedure

Acute oral toxicity was evaluated using OECD Test Guideline 420 as a methodological reference [70], with a modified parallel-group design. Unlike the sequential fixed-dose procedure described in the guideline, four dose levels were evaluated concurrently to characterize clinical observations across a broader range, including 5000 mg/kg. The experiment was therefore not interpreted as a fully guideline-compliant OECD 420 study. Following a 3 h fasting period, six experimental groups (*n* = 5 mice per group) were established. Randomization was performed via a weight-stratified block design using Microsoft Excel software. This procedure ensured that the mean body weight and variance were statistically uniform across all six treatment groups at the start of the study. The control group received 0.9% saline at 10 mL/kg, whereas treatment groups received a single FFAE dose of 300, 1000, 2000, or 5000 mg/kg. The extract was dissolved in saline and administered by oral gavage at 1 mL/100 g.

Animals were observed at 30 min intervals during the first 6 h and subsequently at least once daily for 14 days. Mortality and changes in the skin, fur, eyes, mucous membranes, respiratory and circulatory function, autonomic and central nervous systems, somatomotor activity, and behavior were recorded. Tremors, convulsions, salivation, diarrhea, lethargy, altered sleep, and coma were specifically monitored. Pre-specified humane endpoints included body-weight loss of 20% or more, persistent inability to eat or drink, clinically evident dehydration, and severe weakness or prostration. Body weight was recorded before treatment and on days 7 and 14. At the end of the study, the animals were euthanized using an overdose of pentobarbital sodium.

#### 4.9.3. Statistical Analysis

Data are presented as the mean ± standard error of the mean (SEM). Body weight data were analyzed using two-way repeated-measures ANOVA, with treatment as the between-subject factor and time as the within-subject factor, followed by Dunnett’s multiple-comparison test. Statistical significance was established at *p* < 0.05. For more details, see Supplementary Materials.

## 5. Conclusions

This study integrated HPLC-based phytochemical characterization, compound-level computational hazard screening, an abundance- and bioavailability-weighted mixture framework, uncertainty propagation, preliminary transportability assessment, and acute oral observations of the fresh flower aqueous extract of *C. pentadactylon*. Seventeen metabolites were quantified in the evaluated extract within a non-redundant inventory of 36 experimentally detected or literature-reported compounds. Rosmarinic acid and catechin predominated in the quantified fraction and consequently exerted the greatest influence on the modeled toxicological and protective evidence contributions.

Compound-level screening generated frequent alerts for hERG inhibition, hepatotoxicity, nephrotoxicity, carcinogenicity, and Ames mutagenicity. The normalized five-endpoint TAS classified 23 compounds as having a higher relative alert burden, and 14 compounds were connected to all five endpoints in the primary network. These findings identify priorities for confirmatory evaluation but do not demonstrate toxic potency or toxicity of the complete extract.

The extract-specific profile produced a lower proportional toxicity-evidence index than the literature-informed profile, and this difference remained stable under the uncertainty assumptions evaluated by Monte Carlo analysis. This result supports the importance of using the measured composition of the actual test material rather than assuming that an unweighted literature inventory represents a particular extract. Nevertheless, the ITPM outputs are comparative evidence indices, not calibrated probabilities of toxicity, protection, or safety, and the protective evidence component does not demonstrate neutralization of predicted liabilities.

A single FFAE administration at doses up to 5000 mg/kg produced no mortality, overt clinical toxicity, observable behavioral abnormalities, or statistically significant treatment-related differences in body weight during the 14-day observation period in male CD-1 mice. These findings support low overt acute toxicity under the specific conditions evaluated but do not establish general or long-term safety or exclude subclinical, cardiac, genotoxic, reproductive, or repeated-dose effects.

The principal contribution of this study is an extract-specific framework linking measured phytochemical composition with transparent hazard screening, evidence weighting, uncertainty analysis, and whole-animal observation. Further analytical, toxicokinetic, repeated-dose, histopathological, electrophysiological, and genotoxic evaluation is required to characterize the safety of the extract. Independent datasets with prospectively assigned reference outcomes will also be necessary to calibrate and externally validate the ITPM.

## Figures and Tables

**Table 1:** Phytochemicals reported or experimentally detected in *Chiranthodendron pentadactylon* flowers.

Compound	PubChem CID	Chemical Class	Source
Dacosanol B-1	12620	Long-chain fatty alcohol (aliphatic alcohol)	[38]
Glucose Ester	64689	Carbohydrate derivative/sugar ester	
Octacosene	87821	Long-chain alkene (hydrocarbon)	
Cyanidin 3-Glucoside	441667	Anthocyanin (flavonoid glycoside)	
Tiliroside	5320686	Flavonol glycoside	[39]
Astragalin	5282102	Flavonol glycoside	
Isoquercitrin	5280804	Flavonol glycoside	
Catechin	9064	Flavan-3-ol (flavonoid)	
Epicatechin	72276	Flavan-3-ol (flavonoid)	
Sacarosa	5988	Disaccharide (carbohydrate)	
Gallic Acid	370	Hydroxybenzoic acid (phenolic acid)	
Chlorogenic Acid	1794427	Hydroxycinnamic acid derivative/caffeoylquinic acid	
Syringic Acid	10742	Hydroxybenzoic acid (phenolic acid)	
Vanillic Acid	8468	Hydroxybenzoic acid (phenolic acid)	
P-Hydroxybenzoic Acid	135	Hydroxybenzoic acid (phenolic acid)	
Caffeic Acid	689043	Hydroxycinnamic acid (phenolic acid)	[36]
Ferulic Acid	445858	Hydroxycinnamic acid (phenolic acid)	
p-coumaric acid	637542	Hydroxycinnamic acid (phenolic acid)	
Rutin	5280805	Flavonol glycoside	
Phlorizin	6072	Dihydrochalcone glycoside (flavonoid)	
Myricetin	5281672	Flavonol	
Quercetin	5280343	Flavonol	
Naringenin	439246	Flavanone	
Phloretin	4788	Dihydrochalcone	
Apigenin	5280443	Flavone	
Kaempferol	5280863	Flavonol	
Galangin	5281616	Flavonol	
Carnosol	442009	Phenolic diterpene (abietane diterpenoid)	
Stigmasterol	5280794	Phytosterol (steroid)	
Oleanolic Acid	10494	Pentacyclic triterpenoid (oleanane-type)	
α-amyrin	73170	Pentacyclic triterpenoid (ursane-type)	
β-sitosterol	222284	Phytosterol (steroid)	
Morin	5281670	Flavonol	This study
Gentisic acid	3469	Hydroxybenzoic acid (phenolic acid)	This study
Ursolic acid	64945	Pentacyclic triterpenoid (ursane-type)	This study
Rosmarinic acid	5281792	Hydroxycinnamic acid ester (phenolic acid derivative)	This study

**Table 2:** Frequency of positive endpoint predictions and acute-toxicity classifications.

Endpoint or Toxicity Classification	Compounds	Percentage
Hepatotoxicity	29/36	80.6%
Nephrotoxicity	28/36	77.8%
Mutagenicity	21/36	58.3%
Carcinogenicity	28/36	77.8%
hERG-related cardiotoxicity	33/36	91.7%
Reproductive-effects alert (supplementary)	7/36	19.4%
Higher acute toxicity potential (predicted LD_50_ ≤ 1000 mg/kg)	6/36	16.7%
Intermediate acute toxicity potential (predicted LD_50_ > 1000–2000 mg/kg)	8/36	22.2%
Lower acute toxicity potential (predicted LLD_50_ > 2000 mg/kg)	22/36	61.1%

Note: Both endpoint frequencies and LD_50_-based acute-toxicity classifications were calculated using the complete 36-compound inventory. A lower LD_50_ indicates greater predicted acute oral toxicity. LD_50_ classification does not exclude organ-specific, cardiac, genotoxic, or repeated-dose toxicity.

**Table 3:** Operational categorization of the 36 compounds according to their integrated toxicological alert burden.

Relative Alert Burden	Operational Definition	Compounds	Percentage
Lower	<0.33 of five endpoints positive	1/36	2.8%
Intermediate	0.33 to <0.67	12/36	33.3%
Higher	≥0.67	23/36	63.9%

The predominance of the higher-alert category reflected frequent positive predictions across hepatotoxicity, nephrotoxicity, carcinogenicity, and hERG inhibition. Structural alerts and predicted LD_50_ classifications were retained as supplementary or descriptive evidence and were not counted as additional TAS endpoints.

**Table 4:** Multi-endpoint toxicological profile of the 36 unique phytochemicals identified in *C. pentadactylon* flowers.

Compound	CID	Hepatotoxic	Nephrotoxic	Mutagenic	Carcinogenic	Cardiotoxic	Positive Endpoints	Category	Predicted LD_50_ (mg/kg)	Acute Toxicity Interpretation
Dacosanol B-1	12620	−	−	−	−	+	1/5	Lower	1000	Higher acute toxicity potential
Glucose Ester	64689	+	+	−	−	+	3/5	Intermediate	23,000	Lower acute toxicity potential
Octacosene	87821	+	−	−	+	+	3/5	Intermediate	5050	Lower acute toxicity potential
Cyanidin 3-Glucoside	441667	+	+	+	+	+	5/5	Higher	5000	Lower acute toxicity potential
Tiliroside	5320686	+	+	+	+	+	5/5	Higher	5000	Lower acute toxicity potential
Astragalin	5282102	+	+	+	+	+	5/5	Higher	5000	Lower acute toxicity potential
Isoquercitrin	5280804	+	+	+	+	+	5/5	Higher	5000	Lower acute toxicity potential
Catechin	9064	+	+	−	+	+	4/5	Higher	10,000	Lower acute toxicity potential
Epicatechin	72276	+	+	−	+	+	4/5	Higher	10,000	Lower acute toxicity potential
Sacarosa	5988	−	+	−	−	+	2/5	Intermediate	29,700	Lower acute toxicity potential
Gallic Acid	370	−	+	+	+	+	4/5	Higher	2000	Intermediate acute toxicity potential
Chlorogenic Acid	1794427	−	+	+	+	+	4/5	Higher	5000	Lower acute toxicity potential
Syringic Acid	10742	−	+	+	+	+	4/5	Higher	1700	Intermediate acute toxicity potential
Vanillic Acid	8468	−	+	+	−	+	3/5	Intermediate	2000	Intermediate acute toxicity potential
P-Hydroxybenzoic Acid	135	+	+	+	−	+	4/5	Higher	2200	Lower acute toxicity potential
Caffeic Acid	689043	+	+	+	+	+	5/5	Higher	2980	Lower acute toxicity potential
Ferulic Acid	445858	+	+	+	+	+	5/5	Higher	1772	Intermediate acute toxicity potential
p-coumaric acid	637542	+	+	+	+	+	5/5	Higher	2850	Lower acute toxicity potential
Rutin	5280805	+	+	+	+	+	5/5	Higher	5000	Lower acute toxicity potential
Phlorizin	6072	+	+	+	+	+	5/5	Higher	3000	Lower acute toxicity potential
Myricetin	5281672	+	+	+	+	+	5/5	Higher	159	Higher acute toxicity potential
Quercetin	5280343	+	+	+	+	+	5/5	Higher	159	Higher acute toxicity potential
Naringenin	439246	+	+	−	+	+	4/5	Higher	2000	Intermediate acute toxicity potential
Phloretin	4788	+	+	−	+	+	4/5	Higher	500	Higher acute toxicity potential
Apigenin	5280443	+	+	+	+	+	5/5	Higher	2500	Lower acute toxicity potential
Kaempferol	5280863	+	+	+	+	+	5/5	Higher	3919	Lower acute toxicity potential
Galangin	5281616	+	+	+	+	+	5/5	Higher	3919	Lower acute toxicity potential
Carnosol	442009	+	+	−	+	+	4/5	Higher	1500	Intermediate acute toxicity potential
Stigmasterol	5280794	+	−	−	+	+	3/5	Intermediate	890	Higher acute toxicity potential
Oleanolic Acid	10494	+	−	−	+	+	3/5	Intermediate	2000	Intermediate acute toxicity potential
α-amyrin	73170	+	−	−	+	+	3/5	Intermediate	70,000	Lower acute toxicity potential
β-sitosterol	222284	+	−	−	+	+	3/5	Intermediate	890	Higher acute toxicity potential
Morin	5281670	−	+	+	−	+	3/5	Intermediate	4500	Lower acute toxicity potential
Gentisic acid	3469	+	−	−	+	−	2/5	Intermediate	2000	Intermediate acute toxicity potential
Ursolic acid	64945	+	−	+	−	−	2/5	Intermediate	3919	Lower acute toxicity potential
Rosmarinic acid	5281792	+	+	−	−	−	2/5	Intermediate	5000	Lower acute toxicity potential

## Data Availability

The original contributions presented in this study are included in the article/Supplementary Materials. Further inquiries can be directed to the corresponding author.

## Supplementary Materials

The following supporting information can be downloaded at https://www.mdpi.com/article/10.3390/ph19091354/s1, Figure S1: Register management unit for the conservation of wildlife; Figure S2: Herbarium specimen with the number 181,307; Table S1: Compounds and references found *C. pentadactylon*; Table S2: In silico toxicological evidence and voting framework.

## Abbreviations

The following abbreviations are used in this manuscript:

ADME	Absorption, Distribution, Metabolism, and Excretion
ADMET	Absorption, Distribution, Metabolism, Excretion, and Toxicity
AOT	Acute Oral Toxicity
BAS	Bioavailability Score
BBB	Blood–Brain Barrier
*C. pentadactylon*	*Chiranthodendron pentadactylon*
CID	Compound Identification Number (PubChem Compound Identifier)
CV	Coefficient of Variation
DILI	Drug-Induced Liver Injury
DL	Drug-likeness
GI	Gastrointestinal
hERG	Human Ether-à-go-go-Related Gene
HPS	Heuristic Protective Score
IMSS	Mexican Institute of Social Security
ITPM	Integrative Toxicological Prediction Model
LD_50_	Median Lethal Dose
NAMs	New Approach Methodologies
OECD	Organisation for Economic Co-operation and Development
ProTox-II	Prediction of Toxicity II
QSAR	Quantitative Structure–Activity Relationship
RA	Relative Abundance
ROS	Reactive Oxygen Species
SDF	Structure-Data Format
SMILES	Simplified Molecular Input Line Entry System
SwissADME	Swiss Absorption, Distribution, Metabolism, and Excretion
TCM	Traditional Chinese Medicine
THS	Heuristic Toxicity Score
TPS	Total Protective Score
TTS	Total Toxicity Score
