# Supplementary material for: Predicting the Toxicity In Silico of the Aqueous Extract of Chiranthodendron pentadactylon Flowers. Experimental Evaluation In Vivo
Source: Pharmaceuticals (Basel). 2026 Aug 26;19(9):1354. doi: 10.3390/ph19091354 (PMC13610003; doi:10.3390/ph19091354)
Supplement: Supplementary file 1 [file pharmaceuticals-19-01354-s001.zip › Dunnett table.pdf]

Treatment	Basal<br>Mean±SEM	7 days<br>Mean±SEM	14 days<br>Mean±SEM
Control (NS)	29.24±0.36	31.14±0.52	32.86±1.16
FFAE (300 mg/Kg)	27.63±0.70	31.00±1.10	33.16±0.47
FFAE (1000 mg/Kg)	28.08±0.59	31.04±0.52	32.47±0.51
FFAE (2000 mg/Kg)	31.68±0.09	33.73±0.28	35.23±0.38
FFAE (5000 mg/Kg)	31.73±0.85	33.88±0.88	34.23±0.72

Dunnett's multiple comparisons test	Summary	Adjusted P Value
Basal		
Control (NS) vs. FFAE (300 mg/Kg)	ns	0.3641
Control (NS) vs. FFAE (1000 mg/Kg)	ns	0.6485
Control (NS) vs. FFAE (2000 mg/Kg)	ns	0.0796
Control (NS) vs. FFAE (5000 mg/Kg)	ns	0.0714
7 days		
Control (NS) vs. FFAE (300 mg/Kg)	ns	0.9996
Control (NS) vs. FFAE (1000 mg/Kg)	ns	>0.9999
Control (NS) vs. FFAE (2000 mg/Kg)	ns	0.0544
Control (NS) vs. FFAE (5000 mg/Kg)	ns	0.0594
14 days		
Control (NS) vs. FFAE (300 mg/Kg)	ns	0.9948
Control (NS) vs. FFAE (1000 mg/Kg)	ns	0.983
Control (NS) vs. FFAE (2000 mg/Kg)	ns	0.1348
Control (NS) vs. FFAE (5000 mg/Kg)	ns	0.5827

ns = non-significant
