# Supplementary material for: Predicting the Toxicity In Silico of the Aqueous Extract of Chiranthodendron pentadactylon Flowers. Experimental Evaluation In Vivo
Source: Pharmaceuticals (Basel). 2026 Aug 26;19(9):1354. doi: 10.3390/ph19091354 (PMC13610003; doi:10.3390/ph19091354)
Supplement: Supplementary file 1 [file pharmaceuticals-19-01354-s001.zip › Supplementary data 3.pdf]

Supplementary Data 3

Oscar Salvador Barrera-Vázquez[a], Gil Alfonso Magos-Guerrero[a]*, Juan Luis Escobar-Ramírez[a],

[a] *Department of Pharmacology, Faculty of Medicine, University National Autonomous of Mexico, Postal mail No. 70-297, 04510 Mexico City, Mexico.*

* Corresponding author. *Phone.* +52 55 5623 2165. *E-mail address:* gamagos@unam.mx

MEDIO AMBIENTE
SECRETARÍA DE MEDIO AMBIENTE Y RECURSOS NATURALES

México 2021 Año de la Independencia

Delegación Federal de SEMARNAT
en el Estado de México
Subdelegación de Gestión para la
Protección Ambiental y Recursos Naturales

DFMARNAT/0706/2021
Toluca, México a 24 de febrero de 2021

REGISTRO PARA EL ESTABLECIMIENTO DE UNA UNIDAD DE MANEJO PARA LA CONSERVACIÓN DE LA VIDA SILVESTRE (UMA).

NOMBRE O RAZÓN SOCIAL: C. DAVID GUAJARDO RUZ

Con fundamento en los artículos 27 tercer párrafo de la Constitución Política de los Estados Unidos Mexicanos; 26 y 32 Bis Fracciones I, III, y XXXIX de la *Ley Orgánica de la Administración* Pública Federal; 1, 2 Fracción XXX, 38, 39 y 40 Fracción IX -inciso i- y XIX del Reglamento Interior de la Secretaría de Medio Ambiente y Recursos Naturales; 79, 80, 82, 83, 86, y 87 de la Ley General del Equilibrio Ecológico y la Protección al Ambiente; 9 Fracción XI, 27, 28, 39, 40, 41 y 42 de la Ley General de Vida Silvestre; 12, 30, 31, 32, 33, 34, 35, 36 y Noveno Transitorio, del Reglamento de la Ley General de Vida Silvestre; y en virtud de haber cumplido con los ordenamientos vigentes en la materia, esta Delegación Federal otorga con vigencia INDEFINIDA el REGISTRO a la siguiente UMA:

**NOMBRE DE LA UMA:**	**"FLOR MANITA"**
**UBICACIÓN:**	**PASEO SANTA ANA #142., SAN CARLOS, C.P. 52159, METEPEC, ESTADO DE MÉXICO.**
**SUPERFICIE AUTORIZADA:**	**0.22 HECTÁREAS**
**CLAVE DE REGISTRO:**	**SEMARNAT-UMA-IN-325-MEX/21**
**TENENCIA DE LA TIERRA:**	**PRIVADA**
**TIPO DE PROPIEDAD:**	**PROPIEDAD_X_RENTADA_ EN COMODATO_ P/PODER_ OTRO_**
**FINALIDAD DE LA UMA:**	**REPRODUCCIÓN, EDUCACIÓN AMBIENTAL, , INVESTIGACIÓN, EXHIBICIÓN Y APROVECHAMIENTO EXTRACTIVO**

El presente registro se otorga para la conservación, manejo y aprovechamiento sustentable de la especie: ***Chiranthodendron pentadactylon* (árbol de manitas).** Asimismo se otorga en resguardo un ejemplar de la especie citada con marcaje etiqueta CHPE 1.

Previo a cualquier tipo de aprovechamiento que pretenda realizar de los ejemplares de flora silvestre existentes en la UMA registrada, debe contar con la aprobación del respectivo plan de manejo, estudios poblacionales y solicitarlo en el formato oficial.

Figure S1. Register management unit for the conservation of wildlife.

**DFMARNAT/0706/2021
Toluca, México a 24 de febrero de 2021**

A continuación, se indican las coordenadas UTM de la poligonal de la UMA **"FLOR MANITA".**

VÉRTICE	UTM	
	X	Y
1	2130163.1	414594.68
2	2130181.26	414612.56
3	2130166.05	434631.3
4	2130146.53	434613.42
5	2131163.1	414594.68

ESTE REGISTRO QUEDA SUJETO AL CUMPLIMIENTO DE LAS CLÁUSULAS DE OPERACIÓN ANEXAS, CUALQUIER VIOLACIÓN O INCUMPLIMIENTO DARÁ ORIGEN A LA INSTAURACIÓN DE UN PROCEDIMIENTO ADMINISTRATIVO ANTE LA AUTORIDAD COMPETENTE PARA PROCEDER A LA CANCELACIÓN DEL REGISTRO Y LA APLICACIÓN DE LA NORMATIVIDAD CORRESPONDIENTE, SEGÚN SEA EL CASO.

El presente se emite en apego al principio de buena fe, al que se refiere el artículo 13 de la Ley Federal de Procedimiento Administrativo (LFPA), tomando por verídica la información presentada por el C. David Guajardo Ruz, en su escrito de fecha 18 de febrero del año en curso, en caso de existir falsedad en la información manifestada, el Promovente se hará acreedor a las sanciones correspondientes de acuerdo a lo dispuesto en las fracciones II, IV y V del artículo 420 Quáter del Código Penal Federal, referente a los delitos en materia ambiental.

ATENTAMENTE

SECRETARÍA DE MEDIO AMBIENTE
Y RECURSOS NATURALES
ESTADO DE MÉXICO

ING. JOSÉ ERNESTO MARÍN MERCADO
Con fundamento en lo dispuesto por el artículo 84 del Reglamento Interior de la Secretaría de Medio Ambiente y Recursos Naturales, en suplencia, por ausencia del Titular de la Delegación Federal de la SEMARNAT en el Estado de México[1], previa designación mediante oficio No. 01243 de fecha 28 de noviembre de 2018, firma el presente el Subdelegado de Gestión para la Protección Ambiental y Recursos Naturales

c.c.p.- Ing. Federico Ortiz Flores.- Encargado del despacho de la Delegación de la Procuraduría Federal de Protección al Ambiente en el Estado de México.- Toluca, Méx.

JEMM'MMH'RAAL

Bitácora: 15/U1-0402/02/21

1 *En los términos del artículo 17 Bis en relación con los artículos Octavo y Décimo Tercero Transitorios del Decreto por el que se reforman, derogan diversas disposiciones de la ley Orgánica de la Administración Pública Federal, publicado en el Diario Oficial de la Federación el 30 de noviembre de 2018.*

Figure S1. (*continued*)

Asunto: Constancia de ejemplar botánico.

A quien corresponda

PRESENTE

Por medio de este medio se hace constatar que el ejemplar de Herbario con el numero 181307 colectado en Paseo Sta Ana #142, San Carlos en Metepec, Edo de México, el día 22 de febrero del 2023, corresponde a Chiranthodendron pentadactylon, de la Unidad de Manejo para la Conservación de la Vida Silvestre (UMA) "Flor de Manita" lo asegura permiso SEMARNAT-UMA-IN-325-MEX/21.

Sin existir otro asunto que tratar, aprovecho para enviarle un cordial saludo.

"POR MI RAZA HABLARÁ EL ESPÍRITU"
Ciudad Universitaria a 30 de junio de 2023

Atentamente

Dr. Jaime Jiménez Ramírez
Herbario de la Facultad de Ciencias.
Universidad Nacional Autónoma de México

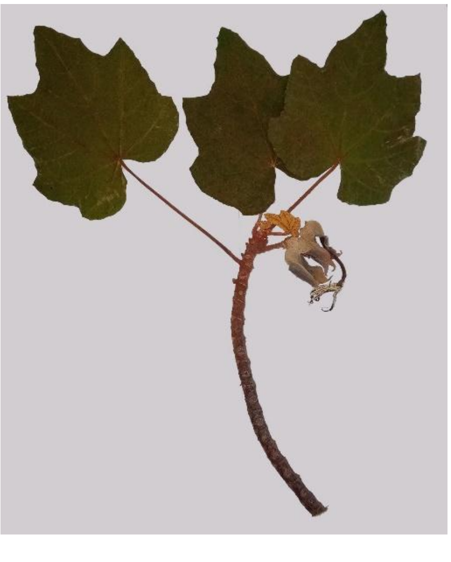

Figure S2. Herbarium specimen with the number 181307.
